# Whole blood RNA sequencing reveals a differential transcriptomic profile associated with cervical insufficiency: a pilot study

**DOI:** 10.1186/s12958-021-00715-2

**Published:** 2021-02-24

**Authors:** Ga-Hyun Son, So Yeon Choi, Yeon-Ji Ju, Keun-Young Lee, Jae Jun Lee, Ji-Eun Song, Youngmi Kim, Sung Taek Park

**Affiliations:** 1 Division of Maternal-Fetal Medicine, Department of Obstetrics and Gynecology, Hallym University College of Medicine, Kangnam Sacred Heart Hospital, 665, Siheung-daero, Youngdeungpo-gu, Seoul, 07442 Republic of Korea; 2 Institute of New Frontier Research, College of Medicine, Hallym University, Hallymdaehak-gil, Chuncheon, 24252 Republic of Korea; 3 Departments of Anesthesiology and Pain Medicine, College of Medicine, Hallym University, Chuncheon, South Korea

**Keywords:** Alpha defensin, Cervical insufficiency, Pregnancy, Preterm birth, RNA-Seq

## Abstract

**Background:**

The uterine cervix is a mechanical and immunological barrier against ascending infection during pregnancy. Cervical insufficiency (CI), a painless cervical dilation that occurs in the mid-trimester, is an important cause of extremely preterm birth. We hypothesized that women with CI have a differential transcriptomic profile. Therefore, we compared the transcriptomic profile of peripheral blood in women with CI and that of controls.

**Methods:**

RNA sequencing was used to generate the global gene expression profiles of 11 women with CI and 4 controls, and differential expression analysis was performed to identify genes showing significant expression changes between the CI (*n* = 11) and control (*n* = 4) groups as well as between the CI-preterm (*n* = 7) and CI-term (*n* = 4) groups. Gene set enrichment was assessed in terms of Gene Ontology processes, and a subset of differentially expressed genes in CI was validated in a different sample-set by qRT-PCR and ELISA.

**Results:**

Thirty genes were differentially expressed between the CI and control groups. Differentially upregulated genes in the CI group included neutrophil-mediated immunity-associated (DEFA3 and ELANE) and bicarbonate transport-related genes. The serum concentration of alpha defensin 3 was significantly higher in women with CI than in controls (*P* = 0.014). Analysis of differential gene expression according to pregnancy outcomes revealed 338 differentially expressed genes between the CI-term and CI-preterm groups. Immune and defense response to organism-associated genes and influenza A and NOD-like receptor signaling pathways were upregulated in the CI-term group.

**Conclusions:**

Our results revealed significant differences in the whole blood transcriptomic profiles of women with CI compared to those of controls. Different immune responses in women with CI may affect pregnancy outcomes.

**Supplementary Information:**

The online version contains supplementary material available at 10.1186/s12958-021-00715-2.

## Background

Preterm birth (PTB), defined as delivery before 37 completed weeks of gestation, occurs in more than one of 10 babies, and approximately one million children die each year because of complications related to PTB [1]. Moreover, prematurity is a huge burden on the healthcare system because of long-term morbidities, such as neurodevelopmental disability as well as immediate complications of prematurity related to organ system immaturity [2–4]. One factor leading to preterm delivery is cervical insufficiency (CI), which is characterized by spontaneous dilation and effacement of the cervix in the absence of painful uterine contractions in the mid-trimester, leading to bulging fetal membranes, premature rupture of the membranes, and, ultimately, expulsion of an immature fetus [5]. CI occurs in approximately 0.5–1% of all pregnancies and is associated with 5–15% of pregnancy losses in the second trimester or extremely PTBs (< 28 weeks) [6, 7]. Although multiple factors, such as cervical instrumentation (e.g., loop electrosurgical excision procedure, dilation and curettage, cold-knife conization, and hysteroscopy), cervical laceration or obstetric injury, collagen disorders (e.g., Ehlers-Danlos), and in utero exposure to diethylstilbestrol, are associated with CI, its etiopathogenesis remains unknown [8]. Thus, CI is diagnosed based on either a classic past obstetric history (≥2 successive prior second- or early third trimester pregnancy losses without preceding uterine contractions) or a combination of obstetric history and measurement of cervical length ) [9]. Moreover, laboratory tests or useful biomarkers for diagnosis or prediction of CI are not available. Cervical cerclage is the treatment of choice for patients with CI; however, the guidelines for determining whether to perform cerclage differ, and even post-cerclage, and there are limitations to predicting the pregnancy outcomes [10]. Thus, identifying gene expression profiles and biological pathways relevant to CI may reveal potential biomarkers or therapeutic targets for CI. RNA sequencing (RNA-Seq) is a technique which uses next-generation sequencing to allow comprehensive transcriptome analyses of genomes [11–13]. Compared to microarrays, RNA-Seq enables non-biased, probe-independent inspection of gene expression data. Therefore, we compared the transcriptomic profile of patients with CI and that of normal controls using RNA-Seq to identify the distinctive gene expression profile in CI. We also examined whether genes were differentially expressed according to the outcomes of pregnancy in women with CI.

## Methods

### Study patients

Patients who presented with minimal or no symptoms in whom speculum examination revealed cervical dilatation with visible fetal membranes in the second trimester were considered to have CI and were included in the study. Cervical dilatation, effacement, and bulging membranes were evaluated by ultrasonography and confirmed by speculum examination. The nitrazine test and/or Actim Prom™ test (Medix Biochemica, Kauniainen, Finland) were performed to rule out premature rupture of membranes. Uterine contractions were assessed by patient perception, cardiotocogram, and abdominal palpation. Patients with cervical dilatation were observed for at least 6 h, and physical examination indicated that cerclage was performed, when preterm labor and chorioamnionitis were excluded. The criteria for the diagnosis of clinical chorioamnionitis include one or more of the following: maternal fever ≥38 °C, maternal or fetal tachycardia (≥100 beats/min and ≥ 160 beats/min, respectively), or uterine tenderness. We excluded women who had fetuses with major malformations, multiple gestations, persistent regular uterine contractions, vaginal bleeding, ruptured membranes, clinical chorioamnionitis, or prior prophylactic cerclage. We also excluded women who had systemic symptoms or signs, such as cough, fever, vomiting, diarrhea, and abnormal skin rash that suggested viral and bacterial diseases that may affect pregnancy; women who had abnormal liver and kidney function test results; and women who had urinary tract infections on urine culture test.

Women in the control group (*n* = 4) were selected among patients who visited the outpatient clinic, without uterine contraction or fluid leakage, ≥3 cm cervical length, and no cervical dilation, confirmed by ultrasonography. To reduce sample heterogeneity, we selected patients with matched maternal and gestational ages. On the day of CI diagnosis, whole blood samples were obtained from the women; for controls, samples were obtained on the day of enrollment in the study. To validate RNA-Seq analysis, qRT-PCR and immunoassay experiments were performed using separate sample-sets of the CI and control groups. Blood samples for qRT-PCR were collected into PAXgene™ RNA stabilization tubes (QIAGEN, Hilden, Germany), part of the blood samples was centrifuged to collect the serum, and both blood and serum samples were cryopreserved at − 80 °C. The study was approved by the ethics committee of our hospital (No. 2017-08-014). Written informed consent to participate in the study was obtained from each individual prior to recruitment.

### Library preparation and sequencing

For RNA-Seq profiling, 5 mL of blood was collected into EDTA tubes. Total RNA was isolated using TRIzol® RNA Isolation Reagents (Life Technologies, Carlsbad, CA, USA) and stored at − 80 °C. The concentration and quality of RNA products were determined on an Agilent 2100 bioanalyzer platform (Agilent Technologies, Santa Clara, CA, USA). mRNA sequencing libraries were prepared with total RNA using the Illumina TruSeq Stranded mRNA Sample Preparation kit (Illumina, San Diego, CA, USA) according to the manufacturer’s protocol. The quality and size of the libraries were assessed using an Agilent 2100 bioanalyzer DNA kit. All libraries were quantified by qPCR using the CFX96 Real Time System (Bio-Rad, Hercules, CA, USA) and sequenced on the NextSeq500 system (Illumina) with a paired-end 75 base pair plus single 8 base pair index run.

### Preprocessing and genome mapping

Potentially existing sequencing adapters and raw quality bases in the raw reads were trimmed by Skewer [14]. The option –x AGATCGGAAGAGCACACGTCTGAACTCCAGTCA and -y AGATCGGAAGAGCGTCGTGTAGGGAAAGAGTGT were used for the common adapter sequence of the Illumina TruSeq adapters, and the option -q 0 -l 25 -k 3 -r 0.1 -d 0.1 was used for trimming low-quality 5′ and 3′ ends of the raw reads. The cleaned high-quality reads after trimming the low-quality bases and sequencing adapters were mapped to the reference genome using STAR software [15]. The strand-specific library option --library-type = fr-firststrand was applied in the mapping process.

### Quantification of gene expression and analysis of differentially expressed genes (DEGs)

We used Cufflinks with the strand-specific library option, −-library-type = fr-firststrand, and other default options to quantify the mapped reads on the reference genome into gene expression values [16]. The gene annotation of the reference genome hg19 from the UCSC genome browser database (https://genome.ucsc.edu) in GTF format was used as the gene model, and the expression values were calculated in fragments per kilobase of transcript per million fragments mapped reads. The DEGs between the CI and control groups, and between the CI-preterm and CI-term groups were analyzed using Cuffdiff software in the Cufflinks package [17]. To compare the expression profiles among the samples, the normalized expression values of the selected DEGs were unsupervised clustered by in-house R scripts. Volcano plots for the expression-fold changes and adjusted *P* values between the two groups were also drawn using in-house R scripts.

### Functional enrichment analysis

To obtain insights into the biological functional role of the differential gene expression between the CI and control groups, gene-set overlapping tests were performed between the DEGs and functionally categorized genes, including biological processes of Gene Ontology, Kyoto Encyclopedia of Genes and Genomes (KEGG) pathways, and other functional gene sets by g:Profiler version 0.6.7 [18]. The Benjamini–Hochberg procedure was used to adjust for multiple testing, and enrichment was defined if the adjusted *P* value was less than 0.05. Multi-dimensional scaling (MDS) was used to examine the efficiency of DEGs associated with CI.

### qRT-PCR validation of DEGs

Total RNA was extracted from the frozen blood samples using a PAXgene Blood RNA Isolation Kit (QIAGEN) according to the manufacturer’s protocol. RNA quality was verified on an Agilent 2100 Bioanalyzer and the RNA quantity was determined using a NanoDrop® ND-1000 spectrophotometer (Thermo Fisher Scientific, Waltham, MA, USA). Genes were selected based on higher statistical significance, greater absolute fold-changes, and clinical significance and were tested by qRT-PCR to confirm the results of RNA-Seq analysis. DEFA3 (defensin alpha 3), JUN (jun proto-oncogene), ELANE (neutrophil elastase), and CD177 (CD177 molecule) were selected for validation experiments between the CI and control groups, whereas CEACAM8 (CEA cell adhesion molecule 8), CRISP3 (cysteine-rich secretory protein 3), DEFA3, and RNASE3 (ribonuclease A family member 3) were selected to confirm the differential expression between the CI-preterm and CI-term groups. Four micrograms of RNA was used for cDNA synthesis with a Maxime RT PreMix Kit (iNtRON Biotechnology, Seoul, South Korea). PCRs were performed using 2× Rotor-Gene SYBR Green PCR Master Mix (QIAGEN) in the Rotor-Gene Q (QIAGEN). Primer sequences are shown in Supplemental Table 1. Actin was used as the housekeeping control. Each sample was run in triplicate, and all samples had standard deviation < 0.5 between the triplicates.

### ELISA for serum alpha defensin 3

The level of serum alpha defensin 3 was determined in duplicate using an ELISA kit (Cusabio, Wuhan, China) according to the manufacturer’s instructions. The concentration of alpha defensin 3 was determined by interpolation from standard curves. The limit of detection assay was 1.87 ng/mL, and the calculated inter- and intra-assay coefficients of variation were < 10%.

### Statistical analyses

Data are shown as the mean ± standard deviation or median (interquartile range) for continuous variables, and as n (%) for categorical variables. Comparisons between the CI and control groups and between the CI-term and CI-preterm group were performed using Mann–Whitney U test. Proportions were compared using Fisher’s exact test. Statistical analyses were conducted using SPSS software (version 23.0, SPSS, Inc., Chicago, IL, USA). *P* < 0.05 was considered as statistically significant.

## Results

### Study subjects

Clinical characteristics of the 15 patients included for RNA-Seq analysis (CI group, *n* = 11; control group, *n* = 4) are shown in Table 1. The maternal and gestational ages at the time of sample collection were not significantly different between the two groups. Four women in the CI group delivered preterm [CI-preterm group; gestational weeks at delivery, 19.8 (17.4–30.9)], and the others were delivered after 37 gestational weeks [CI-term group; gestational weeks at delivery, 38.0 (37.0–39.0)]. White blood cell (WBC) and neutrophil counts of the CI-preterm group were significantly higher than those of the CI-term group (Table 1).

**Table 1:** Clinical characteristics of women with cervical insufficiency and control women

	CI group (*n* = 11)	Controls (*n* = 4)	*P* value	CI-preterm group (*n* = 4)	CI-term group (*n* = 7)	*P* value
Maternal age (years)	34.0 (31.0–37.0)	32.0 (30.3–36.8)	0.66	35.0 (33.3–36.8)	33.0 (29.0–38.0)	0.79
Gestational age at blood collection (weeks)	19.4 (17.3–25.0)	23.0 (19.0–25.2)	0.41	17.8 (16.0–23.3)	21.1 (19.3–25.1)	0.23
Gestational age at delivery (weeks)	37.0 (21.2–38.1)	38.9 (38.2–39.9)	0.04	19.8 (17.4–30.9)	38.0 (37.0–39.0)	< 0.01
Nulliparous, n (%)	4 (36.3)	1 (25.0)	1.00	2 (50.0)	2 (28.6)	0.58
WBC count (cell/mL)	8450 (6880-11,820)	–		12,275 (9293-13,878)	7010 (6540-9080)	0.02
Neutrophil count (cell/mL)	7120 (4620-10,080)	–		10,240 (8055-11,734)	4720 (4600-7120)	0.01
C-reactive protein (mg/L)	4.0 (3.2–5.8)			4.9 (3.4–15.9)	3.9 (2.8–5.5)	0.41
Cervical dilation, n (%)						
≥ 3 cm	7 (63.6)	–		3 (75.0)	4 (57.1)	1.00
< 3 cm	4 (36.4)	–		1 (25.0)	3 (42.9)	1.00

*CI* Cervical insufficiency, *WBC* White blood cell

### DEGs between CI and control groups

**Fig. 1:**
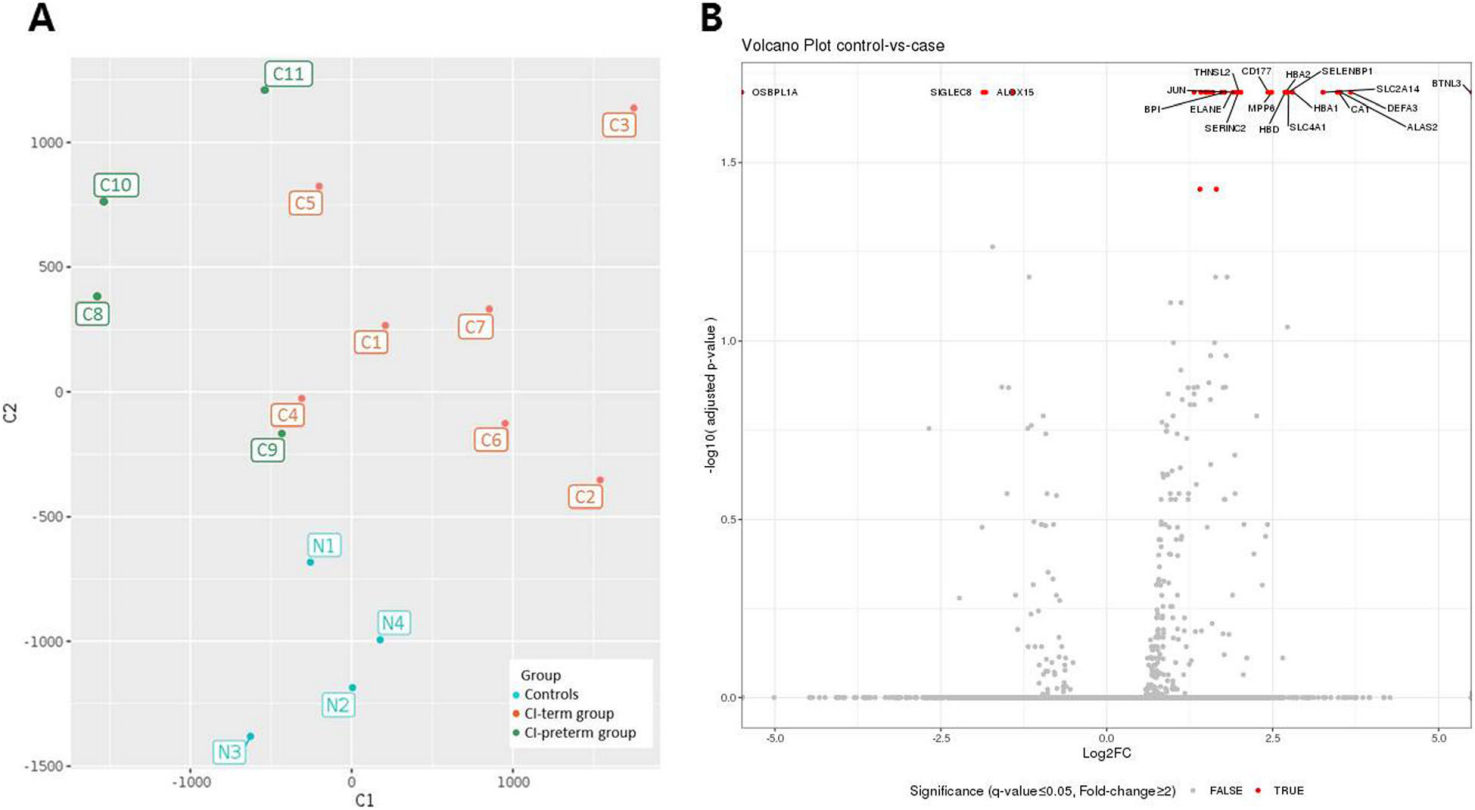
Overall gene expression and differential expression analysis of whole blood transcriptomes. **a** Multi-dimensional scaling (MDS), denoting CI-term group, CI-preterm group, and normal controls. **b** Volcano plot of overall gene-based differential expression results in women with cervical insufficiency vs controls. The x-axis corresponds to the log of the fold change difference between groups and the y-axis corresponds to the negative log of the adjusted *P* values. There were 30 differentially expressed genes (red dots) when using adjusted *P* value < 0.05. CI, cervical insufficiency

**Fig. 2:**
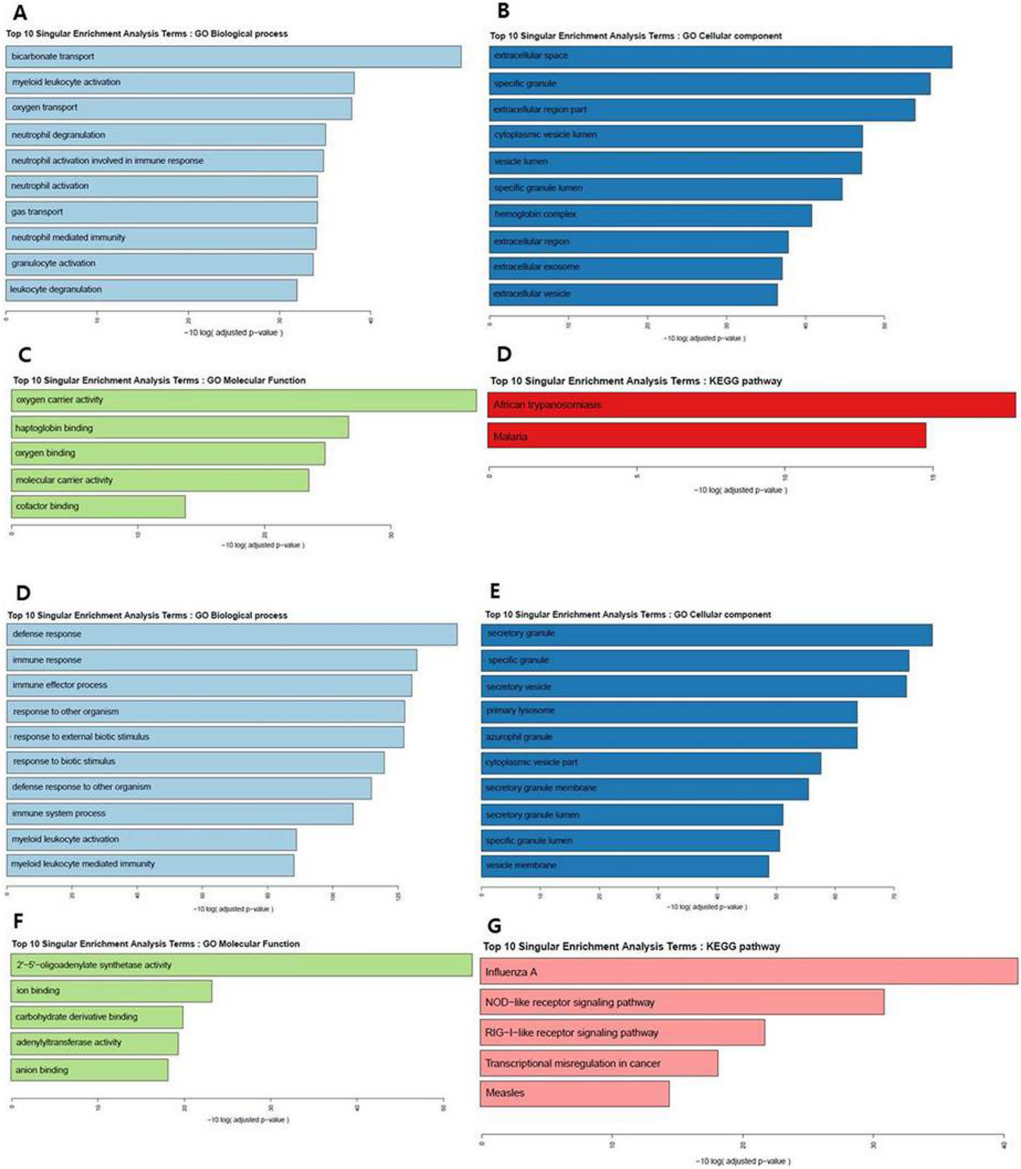
Enriched Gene Ontology (GO) categories (**a–c**) and KEGG pathways (**d**) of genes in cervical insufficiency. GO comparison of biological processes (**a**), cellular components (**b**), and molecular functions (**c**) enriched in genes differentially expressed in women with cervical insufficiency vs controls. Enriched Gene Ontology (GO) categories (**d-f**) and KEGG pathways (**g**) of genes among women with cervical insufficiency who delivered preterm (CI-preterm) vs term (CI-term). GO comparison of biological processes (**a**), cellular components (**b**), and molecular functions (**c**) enriched in genes differentially expressed in CI-term group vs CI-preterm group. All represented GO terms are significantly enriched, with an adjusted *P* value < 0.01. CI, cervical insufficiency

The genome-wide transcriptome in blood samples was analyzed using RNA-Seq for 11 CI and 4 control samples. MDS was performed on gene expression data to determine the similarity pattern between the CI and control groups. The MDS plot showed that control samples were clearly distinguished from the CI samples and that the CI samples presented greater variability (Fig. 1a). Thirty genes were identified as significantly differentially expressed between the CI and control groups (false discovery rate < 0.05, fold-change ≥2) (Fig. 1b, Table 2). Among these, 26 were significantly upregulated and 4 were downregulated in the CI group compared to those in the control group. The upregulated DEGs included neutrophil-mediated immunity-associated genes [DEFA3, CD177, JUN, ELANE, BPI (bactericidal permeability increasing protein), MMP8 (matrix metallopeptidase 8), and CEACAM8)], and bicarbonate transport-related genes [(CA1 (carbonic anhydrase 1), HBA1 (hemoglobin subunit alpha 1), SLC4A1 (solute carrier family 4 member 1)]. To understand the underlying biological pathways that were differentially regulated between the two groups, we performed functional enrichment analysis. The DEGs were enriched in 23 terms in the category of biological processes, and bicarbonate transport, myeloid leukocyte activation, oxygen transport, neutrophil degranulation, and neutrophil activation were the most significantly enriched for the DEGs. Similarly, the DEGs were enriched in 23 terms in the cellular component category and 5 in the molecular function category (Fig. 2a–c). KEGG pathway analysis revealed that the most enriched pathways were malaria and African trypanosomiasis (Fig. 2d).

**Table 2:** Differentially expressed transcripts between CI group and control group

Variable	Symbol	log_2_FoldChange	Adjusted *P* value
Up	DEFA3	3.67511723789818	2.E-02
	ALAS2	3.519309797177	2.E-02
	BTNL3	Inf	2.E-02
	CA1	3.47497905675555	2.E-02
	SLC2A14	3.25605684281671	2.E-02
	HBA1	2.7980605249831	2.E-02
	SELENBP1	2.72166266610296	2.E-02
	SLC4A1	2.71704488249167	2.E-02
	HBA2	2.69537791118106	2.E-02
	HBD	2.68541179635482	2.E-02
	MPP6	2.4833165221545	2.E-02
	CD177	2.42949208713447	2.E-02
	THNSL2	2.02084194732558	2.E-02
	SERINC2	1.98173439063116	2.E-02
	JUN	1.9533367220765	2.E-02
	ELANE	1.90527810295126	2.E-02
	BPI	1.77611402176362	2.E-02
	MMP8	1.72925635901305	2.E-02
	GADD45G	1.65078409116667	4.E-02
	CEACAM8	1.5983927504685	2.E-02
	CRISP3	1.53891127931527	2.E-02
	CEACAM6	1.50022217089997	2.E-02
	PGLYRP1	1.48532447007354	2.E-02
	CHST13	1.41818971026847	2.E-02
	CITED4	1.40404903094954	4.E-02
	CFAP45	1.31521033814004	2.E-02
Down	OSBPL1A	−6.87091809482961	2.E-02
	SIGLEC8	−1.8619380736046	2.E-02
	ALOX15	−1.8254376692933	2.E-02
	IDH3A	−1.417225618276	2.E-02

*CI* Cervical insufficiency

### DEGs between the CI-preterm and CI-term groups

Based on pregnancy outcomes, we further divided the patients in the CI group into CI-term (*n* = 7) and the CI-preterm (*n* = 4) groups and investigated the DEGs between them. Comparison of clinical characteristics between the two groups is shown in Table 1. A total of 338 genes were differentially expressed (false discovery rate < 0.05 and fold-change ≥2) between the CI-term and CI-preterm groups (Supplementary Table 2). Among these, 194 genes were significantly upregulated and 144 were downregulated in the CI-term group compared to in the CI-preterm group. Immune response and defense response to organism-associated genes, such as DEFA3, CXCL10 (C-X-C motif chemokine ligand 10), RNASE3, and CRISP3, were upregulated in the CI-term group, whereas genes involved in myeloid leukocyte-mediated immunity, neutrophil activation, such as FOLR3 (folate receptor gamma), MCEMP1 (mast cell-expressed membrane protein 1), and CD177, were upregulated in the CI-preterm group. Functional enrichment analysis revealed that the DEGs were enriched in 84 terms in the category of biological process, and the terms of defense response and immune response were the most significantly enriched. The DEGs were enriched in 32 terms in the cellular component category and in five terms in the molecular function category (Fig. 2d–f). Further, KEGG pathway analysis revealed that the most significantly upregulated pathways were influenza A, NOD-like receptor (nucleotide-binding oligomerization domain-like receptor), and RIG-I-like receptor (retinoic acid-inducible gene-I-like receptor) signaling pathways (Fig. 2g).

### qRT-PCR validation of DEGs

**Fig. 3:**
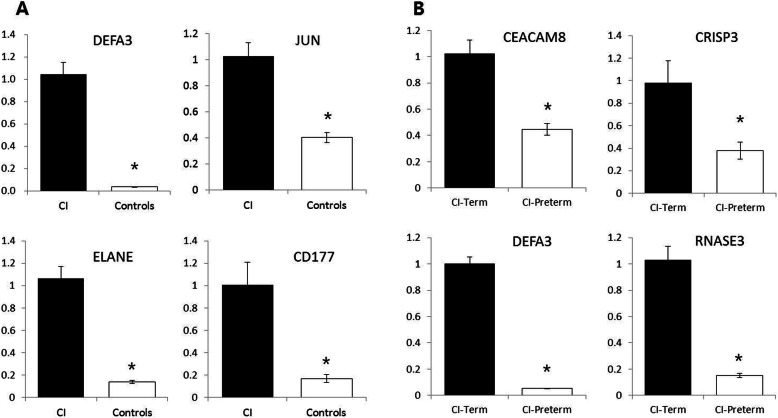
mRNA of selected DEG expression in whole blood of the CI and control groups (**a**) and in whole blood of the CI-term and CI-preterm groups (**b**). * Significant difference (*P* < 0.05) compared with control group. CI, cervical insufficiency

To validate the significant DEGs in the RNA-Seq results, four genes (DEFA3, JUN, ELANE, and CD177) were selected and their differential expression between a separate sample-set of CI (*n* = 11) and control (*n* = 5) groups was assessed by qRT-PCR. Patient demographics and clinical characteristics are presented in Supplementary Table 3. Consistent with the RNA-Seq analysis, four genes displayed higher mRNA expression in the CI group than in the control group (Fig. 3a). We also validated the mRNA expression of the four genes (DEFA3, RNASE3, CRISP3, and CEACAM8) that differed between the CI-term and CI-preterm groups (Fig. 3b). These genes were significantly upregulated in the CI-term group compared to that in the CI-preterm group, and the results were consistent with those of RNA-Seq analysis.

### Alpha defensin 3 expression in women with CI

**Fig. 4:**
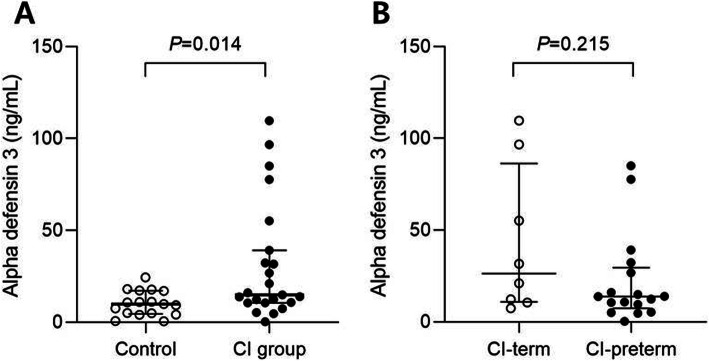
The serum concentration of alpha defensin 3 in the CI and control groups (**a**) and in the CI-term and CI-preterm groups (**b**). CI, cervical insufficiency

RNA-Seq analysis identified DEFA3 as one of the top upregulated DEGs in the CI group compared to in controls; it was also significantly upregulated in the CI-term compared to in the CI-preterm group. Moreover, the DEFA3 mRNA expression pattern was consistent with the results of RNA-Seq analysis. Therefore, we performed an immunoassay to confirm the differential expression of serum alpha defensin 3 in women with CI (*n* = 25) and normal controls (*n* = 17). The clinical characteristics of the patients are shown in Table 3. Alpha defensin 3 was significantly upregulated in the CI group compared to in the control group [14.9 ng/mL (10.5–47.2 ng/mL) vs 9.9 ng/mL (4.6–17.2 ng/mL), *p* = 0.014; Fig. 4a]. We further analyzed the expression of alpha defensin 3 between the CI-term group (*n* = 8) and CI preterm group (n = 17). Although the median concentration of alpha defensin 3 in the CI-term group was higher than that in the CI-preterm group, it was not significant [26.4 ng/mL (11.0–86.3 ng/mL) vs. 14.0 ng/mL (7.4–29.6 ng/mL), *p* = 0.215; Fig. 4b].

**Table 3:** Baseline and clinical characteristics of patients

	CI group (*n* = 25)	Normal controls (*n* = 17)	*P* value	CI-term group (*n* = 8)	CI-preterm group (*n* = 17)	*P* value
Maternal age (years)	34.0 (31.0–37.0)	37.0 (31.5–38.0)	0.20	34.5 (32.3–37.0)	34.0 (30.5–36.5)	0.59
Nulliparous, n (%)	13 (52.0)	6 (35.3)	0.35	2 (25.0)	11 (64.7)	0.68
Gestational age at blood collection (weeks)	22.4 (18.5–25.0)	24.1 (18.0–24.5)	0.33	23.7 (22.3–25.2)	21.4 (19.1–23.8)	0.08
Gestational age at delivery (weeks)	26.4 (21.2–37.3)	38.1 (37.6–38.4)	< 0.01	37.2 (37.0–37.5)	24.2 (20.8–26.9)	< 0.01
Cervical dilation						
≥ 3 cm	18 (72.0)	–		3 (37.5)	15 (88.2)	0.02
< 3 cm	7 (28.0)	–		5 (62.5)	2 (11.8)	0.02
WBC count (cell/mL)	11,680 (9340-14,085)	–		8440 (8250-12,498)	12,970 (10,320-14,280)	0.01
Neutrophil count	9220 (6835-11,495)	–		6375 (5770-9765)	10,150 (7810-12,140)	0.01
C-reactive protein (mg/L)	5.0 (3.9–20.4)	–		4.0 (1.5–4.2)	7.9 (4.7–29.5)	0.01

*CI* Cervical insufficiency

## Discussion

We performed whole blood RNA-Seq in women with CI and compared the transcriptomic profile to that of normal controls. Our results identified upregulation of genes associated with neutrophil activation and neutrophil-mediated immunity in the CI group compared to in the control group. The uterine cervix is positioned between the vagina, with a rich vaginal microorganism environment, and the presumed sterile intrauterine space. Thus, the uterine cervix is implicated as a mechanical barrier against ascending infection by maintaining closure until delivery and an immunological barrier containing immune cells (dendritic cells, neutrophils, and macrophages) and molecular components, including pattern recognition receptors, Toll-like receptors, receptor for advanced glycation end products, cytokines and chemokines, damage-associated molecular patterns, and antimicrobial peptides [19, 20]. Therefore, in women with a disrupted cervical barrier, such as advanced cervical dilation, the risk of intra-amniotic microbial invasion is increased. The upregulated neutrophil immunity-associated genes in women with CI showing clinical manifestations, such as cervical dilation, identified in the present study, presumably protects the fetus by preventing ascending infection. Neutrophils are specialized innate phagocytic cells that are densely packed with granules, which are divided as primary (azurophil), secondary (specific), gelatinase, and secretory granules, and contain several proteins with broad antibacterial action [21, 22]. The granules are heterogenous with respect to their structure, content, and function, and readily released to participate in the host responses to infection or inflammation. In this study, we identified a broad spectrum of neutrophil-related genes expressed at significantly higher levels in women with CI than in controls. Alpha defensins, expressed in azurophil granules, are antimicrobial peptides with remarkable antibacterial, antifungal, and antiviral activities against a wide variety of microorganisms, suggesting their role in innate immunity; they also play important roles in modulating the immune response [23–25]. A previous study reported that maternal plasma defensin levels were increased in women who developed histologic chorioamnionitis after preterm premature rupture of membrane [26]. Therefore, the upregulated DEFA3 expression in the maternal blood of the CI group observed in this study may be explained by the intra-amniotic inflammation that is found in 80% of patients with CI [27].

BPI, a lipopolysaccharide-binding protein with bactericidal activity towards gram-negative organisms, neutralizes lipopolysaccharide-induced pro-inflammatory responses, enhances phagocytosis regulation, and possesses anti-fungal properties [28]. Considering the overexpression of a broad spectrum of neutrophil granule-related genes (ELANE, BPI, MMP8, CRISP3, and CEACAM8) as well as DEFA3 in the CI group, following disruption of the cervical barrier, ascending vaginal microorganism infection may occur, causing intra-uterine inflammation/infection, maternal systemic inflammatory response, and neutrophil activation. Alpha defensin and MMP8 were previously proposed as biomarkers of PTB because of their higher concentrations in the amniotic fluid of PTBs than in that of full-term births [26, 29]. However, there is little information on their expression in CI. Our results showed that the serum concentration of alpha defensin 3 in the CI group was significantly higher than that in controls. Therefore, alpha defensin 3 is a candidate biomarker for CI.

Further, we observed that genes associated with myeloid leukocyte activation and neutrophil-mediated immunity were upregulated in the CI group compared to in the control group, supporting that systemic and intra-uterine inflammation occurs in women with CI. Intra-uterine inflammation may be associated with CI, and its severity is associated with every PTB in women with CI [30]. Our results showed a higher neutrophil count in the CI-preterm group than in the CI-term group, indicating more severe inflammation. Since alpha defensin is produced by neutrophils, it was expected that the concentration of alpha defensin 3 would be higher in the CI-preterm group than in the CI-term group. However, our results showed that the concentration of alpha defensin 3 tended to be higher in the CI-term group. This trend was also observed in the RNA-Seq and qRT-PCR results. DEFA3 expression was higher in the CI-term group than in the CI-preterm group. We speculate the following as the explanation for these results. Although alpha defensins are stored in the azurophil granules of neutrophils, granules normally undergo restricted secretion and are released into the extracellular milieu through degranulation of activated neutrophils. In addition, alpha defensin can be released following apoptosis, necrosis, or neutrophil extracellular trap (NET)-osis of neutrophils [31–34]. Thus, the expression level of alpha defensin is not proportional to the number of neutrophils, but it can be considered to be more related to the degree of activation of neutrophils. For a deeper knowledge on this, further studies, such as examination of neutrophil degranulation markers or serial measurement of defensin levels, are needed. Reduced alpha defensin expression reportedly compromises mucosal host defenses of the ileal mucosa and is associated with the initiation of Crohn’s disease [35]. Moreover, alpha defensins can control inflammation by inhibiting biosynthesis of proinflammatory cytokines by macrophages [32]. In a similar context, our findings showed that the upregulated expression of various host defense-related genes, such as DEFA3, was associated with favorable pregnancy outcomes in women with CI. However, since a high expression of defensin reflects a strong inflammatory response and serves as a double-edged sword through which the host defense mechanism acts, more studies are needed on the effect of DEFA3 gene expression on pregnancy prognosis.

Infection, mainly due to malaria, HIV, and parasites, is known to be a major etiological factor for PTB globally [36]. Consistently, we found that the malaria and African trypanosomiasis pathways were enriched in genes in the CI group. These findings indicate the presence of many underestimated infections in CI that are unidentified by traditional microorganism culture, and current culture techniques are extremely limited as diagnostic tests for infection at the amniotic fluid and systemic levels. Moreover, the pathways we identified as being enriched in genes with the maintenance of pregnancy were NOD-like receptor and RIG-I-like receptor signaling pathways, which are associated with the innate immune response. Therefore, our findings suggest that immunological responses at the maternal–fetal interface and systemic levels play an important role in prolonging pregnancy by modulating inflammatory responses and preventing infection in CI.

To our knowledge, this is the first RNA-Seq study of whole blood in women with CI. To date, there are few high-throughput studies on CI, likely because isolated CI is a relatively rare condition, and there are no objective diagnostic criteria, making it difficult to determine the subject inclusion and obtaining cervical tissue during pregnancy for analysis. In this study, women with a short cervix on ultrasonography were excluded, and only those with cervical dilation were included. The presence of symptoms, such as uterine contractions or vaginal bleeding, was closely observed in the CI group, and patients with any suspected symptoms were excluded from the study group. Moreover, although the pathophysiology or etiology of CI could not be investigated using cervical tissues, our study revealed a systemic unique transcriptomic profile for cervical barrier disruption and differential gene expression according to pregnancy outcomes. Our study had some limitations. First, the sample size was small, particularly in the control group. However, we carefully matched the maternal and gestational ages of women in the CI and control groups, and homogenous gene expression of normal controls enabled us to overcome the limitations of the small sample size. In addition, although the serum alpha defensin 3 concentration in the CI-term group tended to be higher than that in the CI-preterm group, the difference between the two groups was not significant. Thus, studies with a larger sample size are needed to determine the usefulness of serum alpha defensin 3 as a predictive biomarker of pregnancy outcomes. Further, in recent studies, higher levels of beta defensin were observed in the amniotic fluid of patients with spontaneous preterm labor with intraamniotic inflammation than in women without inflammation, and higher vaginal beta defensin levels were also observed to lower the risk of preterm birth [37–40]. Although we found that alpha defensin showed a significant difference between the CI and normal groups, further studies on the expression of other antimicrobial peptides and their effect on pregnancy prognosis are needed to broaden understanding of the pathophysiology of preterm parturition.

## Conclusions

We identified a potential difference in gene expression between the CI and control groups, representing upregulation of neutrophil-activation- and neutrophil-mediated immunity-associated genes in the CI group compared to in the control group. Moreover, differential expression of immune response and defense response to organism-associated genes and the immune response signaling pathway could be associated with pregnancy outcomes in women with CI. Further studies of a larger independent sample are needed to validate the results.

## Supplementary Information

**Additional file 1 **: **Supplementary Table S1.** List of primers used for qRT-PCR.

**Additional file 2 **: **Supplementary Table S2.**

**Additional file 3 **: **Supplementary Table S3.** Clinical characteristics of patients.

## Data Availability

All data generated or analyzed during this study are included in this article and are available from the corresponding author on reasonable request.

## Abbreviations

CI: Cervical insufficiency
GO: Gene Ontology
PTB: Preterm birth
MDS: Multi-dimensional scaling
DEGs: Differentially expressed genes
DEFA3: Defensin alpha 3
RNA-Seq: RNA sequencing
WBC: White blood cell
qRT-PCR: Quantitative RT-PCR
KEGG: Kyoto Encyclopedia of Genes and Genomes
Jun: Jun proto-oncogene
ELANE: Neutrophil elastase
CD177: CD177 molecule
CEACAM8: CEA cell adhesion molecule 8
CRISP3: Cysteine-rich secretory protein 3
RNASE3: Ribonuclease A family member 3
BPI: Bactericidal permeability increasing protein
MMP8: Matrix metallopeptidase 8
CA1: Carbonic anhydrase 1
HBA1: Hemoglobin subunit alpha 1
SLC4A1: Solute carrier family 4 member 1
CXCL10: C-X-C Motif Chemokine Ligand 10
FOLR3: Folate receptor gamma
MCEMP1: Mast cell-expressed membrane protein 1
NOD-like receptor: Nucleotide-binding oligomerization domain-like receptor
RIG-I-like receptor: Retinoic acid-inducible gene-I-like receptor
