# Supplementary material for: Whole blood RNA sequencing reveals a differential transcriptomic profile associated with cervical insufficiency: a pilot study
Source: Reprod Biol Endocrinol. 2021 Feb 24;19:32. doi: 10.1186/s12958-021-00715-2 (PMC7903645; doi:10.1186/s12958-021-00715-2)
Supplement: Supplementary file 1 — Additional file 1 : Supplementary Table S1. List of primers used for qRT-PCR. [file 12958_2021_715_MOESM1_ESM.docx]

Supplementary Table 1. List of primers used for qRT-PCR

| Gene | Sense sequence (5’→ 3’) | Antisense sequence (5’→ 3’) |
| --- | --- | --- |
| DEFA3 | CTTGCTGCCATTCTCCTGGT | ATGCAGGTTCCATAGCGACG |
| JUN | CAAGAACTCGGACCTCCTCA | CTCATCTGTCACGTTCTTGGG |
| ELANE | CACTGCGTGGCGAATGTAA | CTCATCTGTCACGTTCTTGGG |
| CD177 | TGGGCTGTCCACCAAAATGA | TAGCAGGAAGGGCAAACCAC |
| CEACAM8 | GCCTTCACCTGTGAACCTGA | GCACTCGCTGGGTTCTGTAT |
| CRISP3 | AGCTCATGGTCACAAGCAATC | TAGGCATTTCCACATCCAACG |
| RNASE3 | GATGGCGTTGCAAAAACCAAA | AGTAAAGGCACCCGGAATCTA |

***DEFA3:*** defensin alpha 3; ***JUN:*** jun proto-oncogene; ***ELANE:*** neutrophil elastase; ***CD177:*** CD177 molecule; ***CEACAM8:*** CEA cell adhesion molecule 8; ***CRISP3:*** cysteine rich secretory protein 3; ***RNASE3:*** ribonuclease A family member 3.
