# Supplementary material for: Whole blood RNA sequencing reveals a differential transcriptomic profile associated with cervical insufficiency: a pilot study
Source: Reprod Biol Endocrinol. 2021 Feb 24;19:32. doi: 10.1186/s12958-021-00715-2 (PMC7903645; doi:10.1186/s12958-021-00715-2)
Supplement: Supplementary file 3 — Additional file 3 : Supplementary Table S3. Clinical characteristics of patients. [file 12958_2021_715_MOESM3_ESM.docx]

Supplementary Table 3. Clinical characteristics of patients

|  | CI group (n=11) | Controls (n=5) | P value |
| --- | --- | --- | --- |
| Maternal age (years) | 33.0 (32.0-34.0) | 35.0 (32.0-40.0) | 0.22 |
| Gestational age at blood collection (weeks) | 21.4 (18.5-24.3) | 16.4 (15.2-20.4) | 0.04 |
| Gestational age at delivery (weeks) | 37.5 (25.3-38.0) | 38.2 (38.1-39.8) | <0.01 |
| Nulliparous, n (%) | 3 (27.3) | 2 (40.0) | 1.00 |
| Cervical dilation, n (%) |  |  |  |
| ≥ 3 cm | 4 (36.4) | - |  |
| < 3 cm | 7 (63.6) | - |  |

***CI***: Cervical insufficiency.
